# Reproducibility of echocardiographic indices of left atrial size in dogs with subclinical myxomatous mitral valve disease

**DOI:** 10.1111/jvim.15850

**Published:** 2020-07-09

**Authors:** Weihow Hsue, Lance C. Visser

**Affiliations:** ^1^ Department of Medicine & Epidemiology School of Veterinary Medicine, University of California Davis California USA

**Keywords:** canine, echocardiography, endocardiosis, left atrium, preclinical, reliability, repeatability

## Abstract

**Background:**

Reliability of echocardiographic measurements of left atrial (LA) size, an important marker of disease severity, has not been reported in dogs with myxomatous mitral valve disease (MMVD).

**Objectives:**

To define and compare reliability of left atrial dimension/diameter (LAD), LAD indexed to aortic valve diameter (LAD/AoD), left atrium‐to‐aortic root ratio (LA/Ao), left atrial volume acquired from a right parasternal long‐axis (LAV^RPLx^), and left apical view (LAV^LAP^) in dogs with subclinical MMVD.

**Animals:**

Nine dogs with subclinical MMVD.

**Methods:**

Prospective reproducibility study. Dogs underwent 12 echocardiographic examinations by 2 operators on the mornings and afternoons of 3 nonconsecutive days within 1 week. Reliability (measurement variability) was quantified using coefficients of variation (CV) and 95% repeatability/reproducibility coefficients (95% RC). A mixed‐model analysis of variance (ANOVA) was used to determine if time of day, day, and operator were significant sources of variability for each index.

**Results:**

Linear measurements (LAD, LAD/AoD, and LA/Ao) exhibited less within‐day, between‐day, and interoperator variability (CVs, 3.9%‐12.5%) than did volume estimate measurements (LAV^RPLx^ and LAV^LAP^; CVs, 11.8%‐17.9%). Of the linear measurements, LA/Ao exhibited greater variability (CVs, 9.9%‐12.5%) compared to LAD and LAD/AoD (CVs, 3.9%‐4.9%). Operator was a significant (*P* = .005) source of variability for LA/Ao.

**Conclusions and Clinical Importance:**

Compared to other linear measurements, LA/Ao was the least reproducible and most dependent on operator. The 95% RC for each LA size index are provided to help identify clinically relevant changes (beyond intraoperator or interoperator variability) during serial echocardiographic examinations of dogs with subclinical MMVD.

**Abbreviations**
Ao: aortic root
AoD: aortic valve diameter
CV: coefficient of variation
ICC: intraclass correlation coefficient
LA: left atrium/atrial
LAD: left atrial dimension/diameter
LAP: left apical imaging plane
LAV: left atrial volume
MMVD: myxomatous mitral valve disease
RC: repeatability/reproducibility coefficient
RPLx: right parasternal long‐axis imaging plane
*r*_s_: Spearman correlation coefficient
wSD: within‐subject SD

## 1 INTRODUCTION

Myxomatous mitral valve disease (MMVD) is the most common cardiac disease and most frequent cause of heart failure in dogs. Progressive mitral valve regurgitation results in enlargement of the left atrium (LA) and ventricle. Assessment of LA size represents an especially important marker of disease severity. Echocardiographic indices of LA size have been shown to predict risk for heart failure,^1^, ^2^ guide monitoring and treatment during the subclinical period,^3^, ^4^, ^5^, ^6^ and impact prognosis in dogs with MMVD.^3^, ^4^, ^7^, ^8^, ^9^, ^10^

Quantitative LA size assessment most commonly is performed using linear measurements derived from 2‐dimensional echocardiography,^11^, ^12^ but volume estimates using 2‐dimensional and 3‐dimensional echocardiography also have been evaluated for clinical use.^7^, ^13^, ^14^, ^15^, ^16^, ^17^, ^18^, ^19^, ^20^, ^21^, ^22^ Linear measurements are simple and efficient, but are crude surrogates of chamber volume. Conversely, volume estimate measurements that utilize planimetry or 3‐dimensional echocardiography are more representative of true chamber volume, but are less efficient and can be more challenging to measure. Thus, they might be less precise and reproducible. Additionally, 3‐dimensional echocardiography technology currently is hindered by accessibility, transducer size limitations, and cost.

The most commonly reported measurement of LA size is the LA‐to‐aortic root ratio (LA/Ao), acquired from a single right parasternal short‐axis image. It provides a convenient body size‐independent measurement of LA size and is typically the representative echocardiographic index of LA size reported in studies of dogs with MMVD.^4^, ^5^, ^6^, ^8^ However, potential limitations of this method include incorporating a pulmonary vein in the LA measurement,^23^ lack of consistency of timing of the measurements within the cardiac cycle,^24^, ^25^ and defining the path of aortic root measurement relative to valve sinuses.

One alternative linear measurement of LA size is the left atrial diameter/dimension (LAD) acquired from a standard right parasternal long‐axis 4‐chamber image. It can be normalized to body size using an allometric equation^21^, ^25^, ^26^ or indexed to the aortic valve diameter (AoD) acquired from a separate standard right parasternal long‐axis image of the left ventricular outflow view.^21^, ^27^ Doing so avoids incorporating a pulmonary vein, avoids measuring a sinus of Valsalva, and standardizes timing of the measurements for LAD (just before opening of the mitral valve) and AoD (during early to midsystole between the hinge points of the maximally opened aortic valve cusps).

The ideal LA size measurement used for clinical practice should be accurate, precise, and reproducible. Studies assessing accuracy (ie, comparing to a recognized gold standard such as magnetic resonance imaging) are challenging and often absent. This absence underscores the importance of studies assessing precision and reproducibility, which are of great importance and have clinical relevance.^28^, ^29^ Delineating true change in indices of LA size caused by disease progression or regression rather than change related to measurement variation, physiological variability, or both is clinically valuable. Also, assessing the reproducibility of LA size measurement bears relevance to future clinical studies and trials that involve serial echocardiographic examinations, multiple sonographers, or both. For example, measurements with higher reproducibility (less measurement variation) provide greater statistical power to detect differences between groups, which decreases sample size and cost.^30^

To our knowledge, reproducibility of several linear and volume estimate measurements of LA size have not been evaluated in dogs with clinically stable MMVD. Therefore, our objective was to evaluate and compare both intraoperator and interoperator reliability (ie, magnitude of error and variability between measurements) of several linear and volume estimate measurements of LA size in dogs with subclinical MMVD.

## 2 MATERIALS AND METHODS

All procedures in this study were approved by the Institutional Animal Care and Use Committee at the University of California, Davis (protocol #: 20438). All dog owners provided written, informed consent before enrollment.

### 2.1 Animals

Dogs diagnosed with subclinical MMVD and having a complete echocardiographic examination by the clinical cardiology service at our hospital were prospectively recruited for the study. To be eligible for inclusion, dogs had to be ≥6 years of age, have a body weight ≤20 kg, have a characteristic left apical systolic murmur grade ≥3 of 6, and be free of clinical signs. Echocardiographic examinations were reviewed for the presence of mitral regurgitation using color Doppler imaging and valve thickening or irregularity, leaflet prolapse or both. Dogs were excluded if they were affected with any other cardiovascular disease, if they were receiving medications known to affect the cardiovascular system, if their temperament was not conducive for multiple echocardiographic examinations, or if they had previous or current radiographic and clinical evidence of heart failure.

### 2.2 Experimental design

After thoracic radiographs to confirm dogs were not in left heart failure, 9 dogs underwent repeated echocardiographic examinations by 2 operators (W. H. and L. C. V.) on the mornings and afternoons of 3 nonconsecutive days of 1 week (eg, Monday, Wednesday, Friday).^31^ Each dog underwent 12 echocardiographic examinations (6 per operator) for study purposes. The morning and afternoon sessions were at least 3 hours apart. The sequence of operator for a given dog during any session (morning or afternoon) was determined randomly. Medications considered to be necessary for management of subclinical MMVD (eg, pimobendan) were withheld until study completion. Both operators performed their own measurements and were masked from their previous measurements and the measurements of the other operator.

### 2.3 Echocardiographic examinations

#### 2.3.1 Image acquisition

Echocardiographic examinations were performed by a cardiology resident in training at a busy teaching hospital and a board‐certified cardiologist. The cardiologist participates in some of the resident's training. The same ultrasound unit (Philips EPIQ 7C, Philips Healthcare, Andover, Massachusetts) equipped with several phased‐array transducers that were matched to the size of the dog was used. Simultaneous ECG was utilized. Recommended tomographic imaging planes were utilized^32^ including a right parasternal long‐axis 4‐chamber view optimized for the LA and mitral valve, a right parasternal long‐axis view optimized for the left ventricular outflow tract and visualization of the aortic valve cusps, a right parasternal short‐axis basilar view optimized for the LA and visualization of the commissures of the aortic valve cusps in diastole, and a left apical 4‐chamber view optimized for the left heart. Care was taken to avoid foreshortening of the cardiac chambers. At least 6 cardiac cycles from each imaging plane were acquired. Dogs were restrained manually in right and left lateral recumbency. Dogs were not sedated. Recordings from each study were captured digitally for off‐line analysis, which was performed using dedicated software (Syngo Dynamic Workplace, Siemens Medical Solutions, Inc, Malvem, Pennsylvania) at an off‐cart work station.

#### 2.3.2 Echocardiographic measurements

The value recorded for each measurement consisted of the average of 3, usually consecutive, cardiac cycles. No attempts to standardize heart rate or respiratory rate were made during image acquisition or measurements. For all cardiac chamber measurements, the blood‐tissue interface (ie, inner edge‐to‐inner edge) measurement technique was utilized. To avoid precision bias, investigators did not specifically discuss how to perform the measurements. Instead, investigators performed all measurements as they would on clinical patients, using cited veterinary literature^12^, ^17^, ^18^, ^21^, ^27^ as a guide. Measurements performed were maximum LAD^21^, ^27^ and maximum left atrial volume from the right parasternal long‐axis 4‐chamber view (LAV^RPLx^),^21^ aortic valve annulus diameter (AoD) in systole from the right parasternal long‐axis view optimized for the LV outflow tract,^21^, ^27^ LA and aortic root (Ao) from the right parasternal short‐axis basilar view,^12^ and maximum left atrial volume from the left apical 4‐chamber view (LAV^LAP^).^17^, ^18^ Monoplane Simpson's method of discs was used to estimate left atrial volumes. All measurements were performed using 2‐dimensional echocardiography. Measurements of left atrial size were indexed (normalized) to body size as follows: LAD (cm/kg^0.309^),^21^ LAD/AoD, LA/Ao, LAV^RPLx^ (mL/kg), and LAV^LAP^ (mL/kg). Both operators routinely perform LAD, LAD/AoD, and LA/Ao measurements on clinical patients. The LAV^RPLx^ and LAV^LAP^ are measured less frequently and on a case‐by‐case basis.

### 2.4 Statistical analysis

Statistical analyses were performed using computer software (MedCalc Statistical Software, MedCalc Software bvba, Ostend, Belgium and R statistical computing, R package version 0.84.1, Vienna, Austria). Reliability of the repeated echocardiographic studies was quantified using coefficients of variation (CV) and 95% repeatability/reproducibility coefficients (95% RC), both of which utilize the within‐subject SD (wSD). The wSD was calculated as the square root of the within‐subject variance (mean square error), which was determined by 1‐way ANOVA with dogs as the grouping variable. When multiple ANOVAs were utilized, the within‐subject variance was averaged to provide a representative value for calculations of CV and 95% RC. Coefficients of variation were determined using the wSD method and were calculated as: (wSD ÷ overall mean) × 100. The 95% RC were calculated as 1.96 × √2 × wSD.^30^ To determine if operator, time of day, or day were significant sources of variation for each LA size measurement, mixed‐model ANOVAs were performed using dog as a random effect and operator, time of day, and day as fixed effects. Spearman rank correlation coefficients (*r*_s_), intraclass correlation coefficients (ICC), and mean (SD) bias (using Bland‐Altman's method) also were determined for interoperator reproducibility assessments. For ICC calculations, a 2‐way single measures mixed effect model (all subjects are measured by the same observers) for absolute agreement was selected.^33^ Statistical significance was set at *P* < .05.

## 3 RESULTS

Mean (SD, minimum‐maximum) body weight of the dogs enrolled in the study was 7.8 (3.1, 4.2‐14.6) kg. Mean (SD, minimum‐maximum) age was 10.2 (2.5, 6.0‐13.7) years. Eight dogs were spayed females and 1 was a castrated male. Five were mixed breed, 2 were Cavalier King Charles Spaniels, and 1 each were a Boston Terrier and Border Collie. When measurements from each of 12 echocardiographic examinations were averaged, 3 of the 9 dogs had left atrial enlargement when defined by LA/Ao >1.6^5^ (specifically, 1.75, 1.69, and 1.66).

Within‐day, between‐day, and interoperator reliability are summarized in Table 1. All CVs were <20%, and except for between‐day and interoperator reliability of LAV^LAP^, all CVs were <15%. The linear measurements LAD and LAD/AoD had the least variability and had CVs <5%. The CVs for LA/Ao ranged from 9.9% to 12.5%, with highest CV for interoperator variability. The volume estimate measurements of LA size (LAV^RPLx^ and LAV^LAP^) had the highest variability, with CVs ranging from 11.8% to 17.9%. The 95% RC that represent variation from within‐day, between‐day and interoperator variability also are presented in Table 1. In general, 95% RC that represent interoperator variability were slightly higher than intraoperator variability (within‐day and between‐day).

**TABLE 1:** Reliability of left atrial size indices from repeated echocardiographic examinations of 9 dogs with subclinical myxomatous mitral valve disease

	Within‐day (intraoperator)		Between‐day (intraoperator)		Interoperator	
Left atrial size index	CV (%)	95% RC^a^	CV (%)	95% RC^a^	CV (%)	95% RC^a^
LAD (cm/kg^0.309^)	4.3	0.18	3.9	0.18	4.7	0.20
LAD/AoD	4.9	0.33	4.4	0.30	4.9	0.33
LA/Ao	9.9	0.41	10.0	0.44	12.5	0.51
LAV^RPLx^ (mL/kg)	12.5	0.60	11.8	0.59	13.7	0.66
LAV^LAP^ (mL/kg)	14.6	0.60	17.5	0.66	17.9	0.67

Abbreviations: Ao, aortic root; AoD, aortic valve diameter; CV, coefficient of variation; LA, left atrium; LAD, left atrial dimension/diameter; LAP, left apical imaging plane; LAV, left atrial volume; RC, repeatability/reproducibility coefficient; RPLx, right parasternal long‐axis imaging plane.

a 95% RC is in the same unit as the left atrial size index.

**FIGURE 1:**
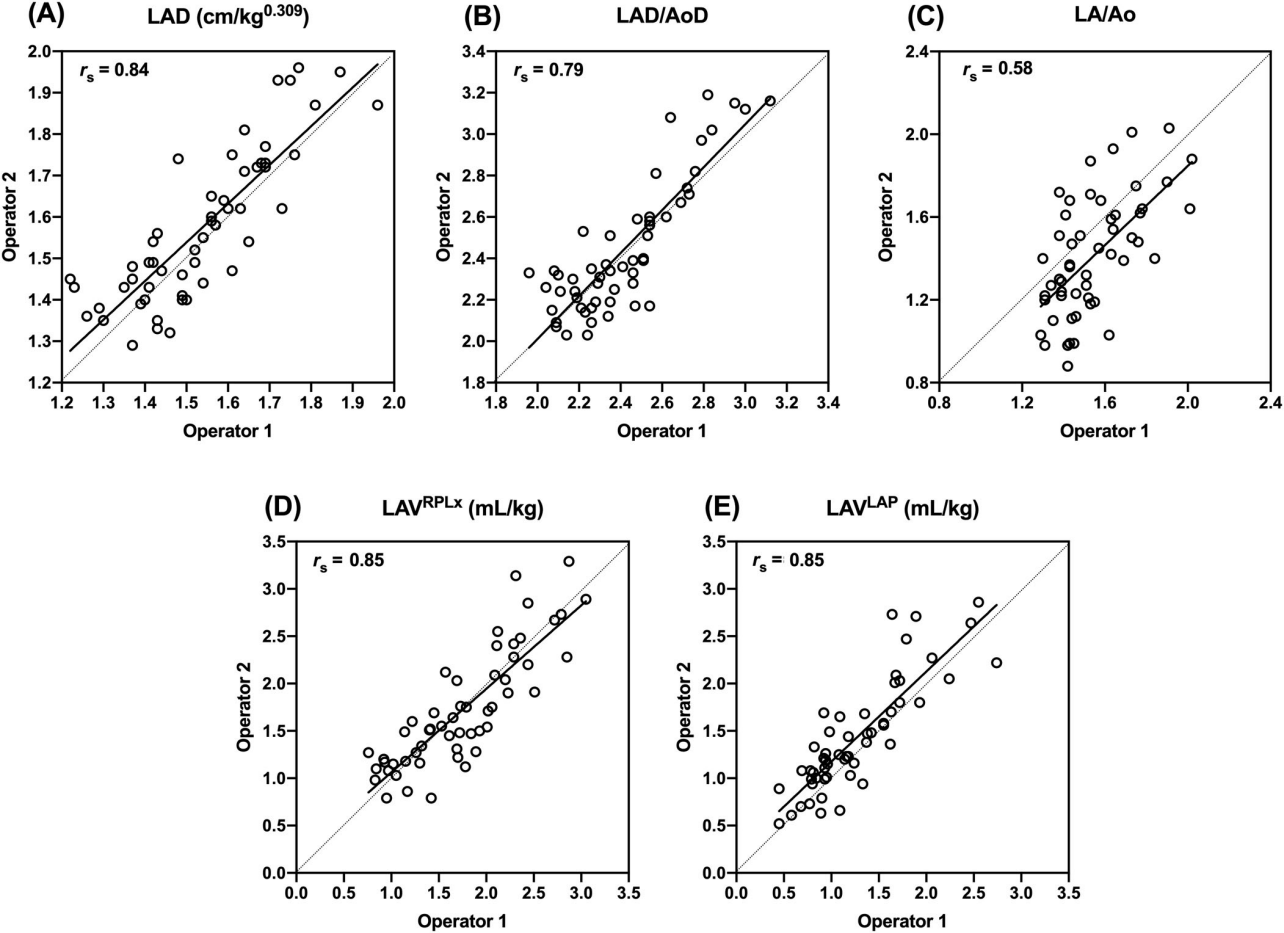
Scatter plots showing the reproducibility assessments between two operators for A, left atrial dimension (LAD); B, LAD indexed to aortic valve diameter (LAD/AoD); C, left atrium to aortic root ratio (LA/Ao); D, left atrial volume acquired from the right parasternal long‐axis view (LAV^RPLx^); and E, left atrial volume acquired from the left apical 4‐chamber view (LAV^LAP^). Nine dogs with subclinical myxomatous mitral valve disease had 12 echocardiographic examinations (6 per operator) performed over 1 week. The dashed line represents the line of equality (perfect agreement) and the solid (black) line represents the line of best fit. Spearman correlation coefficients (*r*_s_) are also presented

The mixed‐model ANOVA showed that operator was a significant (*P* = .005) source of variability for LA/Ao. Significant sources of variability (time of day, day, or operator) were not identified for LAD, LAD/AoD, LAV^RPLx^, and LAV^LAP^ (*P* ≥ .1). Scatter plots illustrating interobserver reproducibility assessments for all LA size indices are presented in Figure 1. Spearman (*r*_s_) and ICC and mean (SD) bias for the interobserver reproducibility analysis of the LA size indices are presented in Table 2. Relatively strong correlations and agreement were observed between operators for LAD, LAD/AoD, LAV^RPLx^, and LAV^LAP^ (*r*_s_ ≥ 0.79, ICC ≥ 0.82, respectively), whereas moderate correlation and agreement were observed between operators for LA/Ao (*r*_s_ = 0.58, ICC = 0.49, respectively). Considerable bias was noted between operators for LA/Ao, where operator 1 tended to measure larger LA sizes compared to operator 2.

**TABLE 2:** Interoperator reproducibility assessment of left atrial size indices from repeated echocardiographic examinations of 9 dogs with subclinical myxomatous mitral valve disease

Left atrial size index	Spearman correlation coefficient (95% CI)	Intraclass correlation coefficient (95% CI)	Mean bias^a^ (SD)
LAD (cm/kg^0.309^)	0.84 (0.74‐0.90)	0.82 (0.70‐0.90)	0.04 (0.10)
LAD/AoD	0.79 (0.66‐0.87)	0.84 (0.74‐0.90)	0.03 (0.17)
LA/Ao	0.58 (0.37‐0.73)	0.49 (0.19‐0.69)	−0.13 (0.23)
LAV^RPLx^ (mL/kg)	0.85 (0.75‐0.91)	0.85 (0.75‐0.91)	−0.03 (0.34)
LAV^LAP^ (mL/kg)	0.85 (0.76‐0.91)	0.82 (0.63‐0.90)	0.16 (0.30)

Abbreviations: Ao, aortic root; AoD, aortic valve diameter; CI, confidence interval; LA, left atrium; LAD, left atrial dimension/diameter; LAP, left apical imaging plane; LAV, left atrial volume; RPLx, right parasternal long‐axis imaging plane.

a Mean bias is in the same unit as the left atrial size index.

## 4 DISCUSSION

Our results showed that linear measurements exhibited less variability (when quantified by CV) than did the volume estimate measurements when evaluated over time (within 1 week) by the same operator and by different operators. Of the linear measurements, LA/Ao exhibited higher variability compared to LAD and LAD/AoD, which were similar. Of the volume estimate measurements, LAV^RPLx^ exhibited less variability compared to LAV^LAP^. When the effects of time (within‐day and between‐day) and operator were evaluated as sources of variability for each of the 5 indices of LA size, only LA/Ao was shown to be significantly impacted by operator. The 95% RC for each LA size index were provided to help identify clinically relevant changes (beyond intraoperator or interoperator variability) during serial echocardiographic examinations of dogs with subclinical MMVD.

The terminology and statistical methods used to quantify measurement variation of echocardiographic measurements can be overwhelming, confusing, and even misleading. We reviewed current and relevant echocardiography literature to help clarify terminology and guide our interpretation and statistical methods.^28^, ^29^, ^34^ Reproducibility is a broad term that represents the variation of the same measurement made on a subject under changing conditions such as different operators, locations, instrumentation, environments, or time frames.^29^, ^30^ Repeatability represents variation in repeated measurements made on the same subject under identical conditions.^29^ It largely represents a test‐retest procedure within a short time frame (eg, a few minutes), ideally under similar hemodynamic conditions.^29^ Reliability represents the magnitude of error or variability between measurements.^29^ Reproducibility, repeatability, and reliability can be assessed by many statistical tests that evaluate correlation, association, bias, agreement, and magnitude of error between measurements, and each statistical test has advantages and disadvantages and might be misleading depending on the type of assessment and data gathered.

Our study consisted of a reproducibility assessment of several echocardiographic indices of LA size and evaluated the variability of the indices over time and by different operators. Subjects, instrumentation, environment, and location did not change. We chose to quantify the reliability of LA size measurements using CV,^29^ which represents the ratio of the SD (within‐subject) to the mean. This coefficient is popular in the veterinary echocardiography literature. It is a dimensionless index that permits comparisons among different indices or measurements within the same study. However, broad applicability (ie, comparisons to other studies and direct relevance to clinical practice) is limited.^31^ This is largely because of the CV's dependence on the overall mean, which inevitably will vary among studies and clinical contexts. Although smaller percentages indicate more precise measurements, acceptable limits for CV (eg, <10% or 20%) are arbitrary and their basis is unclear.

In light of these issues with CV, we also report 95% RC for each LA size index. The 95% RC (also called British Standards Institute value^35^, ^36^) estimates the limits (in the same units as the measurement of interest) between which a repeated measurement is expected to fall with 95% confidence. It assumes no true change in the measured variable caused by, for example, disease progression or regression. Although 95% RC does not readily permit comparisons among different indices (because of different units and scales), it does permit comparisons among different studies of the same measurement or index. It also can help determine what difference likely represents a clinically relevant change in an echocardiographic measurement. For example, our results suggest that in order to be certain (with 95% confidence) that LA size truly has increased when measured using LA/Ao (beyond measurement variability), it must increase by 0.44 (eg, increase from 1.30 to 1.74) when measured by the same operator on a different day and increase by 0.51 (eg, increase from 1.3 to 1.81) when measured by a different operator. Between‐day, intraoperator 95% RC from several of the same LA size indices recently were reported in a study using healthy dogs.^21^ With the exception of LAV^RPLx^, results were similar when comparing the 95% RC from 10 healthy dogs (examined on 2 different days) to those of our study: LAD, 0.10 vs 0.18 cm/kg^0.309^; LAD/AoD, 0.27 vs 0.30; LA/Ao, 0.44 vs 0.44; and, LAV^RPLx^, 0.26 vs 0.59 mL/kg.

Our results follow the intuitive hypothesis that, as the complexity of the measurement increases, reliability decreases. Left atrial dimension/diameter requires only a single linear measurement to quantify LA size, whereas LAD/AoD and LA/Ao require 2 linear measurements. Volume estimate measurements require manually tracing the entire LA internal border and thus permit more opportunity for error, variability, or both. Also, the operators had less clinical experience performing the LAV^RPLx^ and LAV^LAP^ measurements, which also could have influenced the results to some extent. This factor does not suggest volume estimates should be abandoned for assessment of LA size. A recent study showed that LA volume estimates from 2‐dimensional echocardiographic images are accurate surrogates of LA volume in healthy dogs when compared to ECG‐gated multidetector computed tomography, a volumetric gold standard.^37^ Also, several studies^14^, ^18^, ^22^ have documented significant discrepancies when volume estimates are compared to linear measurements (LA/Ao) of LA size in dogs with MMVD. This is presumably because LA volume estimates are more accurate and apt to identify LA enlargement. Thus, clinicians must consider accuracy, precision and reproducibility when selecting indices of LA size for clinical use or for use in future clinical research studies.

When comparing LA/Ao to a directly comparable metric within our study—LAD/AoD (both require 2 linear measurements; 1 of the LA and 1 of the aorta)—LA/Ao was considerably less reproducible. A study^21^ in healthy dogs had similar findings. We suspect this result is a consequence of the aforementioned challenges in standardizing this measurement (ie, the issues previously raised about LA/Ao). Our study suggests this outcome is particularly true among different operators. The LA/Ao index was the only index for which operator was shown to be a significant source of variability. The other indices evaluated appear to be less affected by operator (and time). Our interoperator reproducibility assessment (Table 2 and Figure 1) indicates considerable bias and only moderate correlation and agreement for LA/Ao compared to minimal bias and strong correlations and agreement for the other LA size indices evaluated. This finding is clinically relevant and suggests the same sonographer (and the same person performing measurements) should be utilized for serial evaluations of LA size when using LA/Ao. This guideline might be less important when using LAD, LAD/AoD, LAV^RPLx^ or LAV^LAP^. These results also are relevant to future clinical studies that utilize LA/Ao for LA size assessment and multiple sonographers. The significant interoperator variability of LA/Ao might limit its statistical power to detect differences among groups, thus requiring larger sample sizes and increased cost of clinical studies.

Our study had some limitations. We evaluated precision (reproducibility) of echocardiographic indices of LA size, not accuracy. The accuracy of all of these indices in dogs with MMVD cannot be compared and deserves further study. Our study only evaluated dogs with relatively mild MMVD. Results might differ across a wider spectrum of severity of MMVD and LA sizes. However, measurement variability data is perhaps most relevant to dogs in the subclinical stage (ACVIM stage B) where, currently, the identification of mild LA enlargement is relevant to decisions on starting life‐long medication(s).^4^, ^5^, ^6^ Therapeutic decisions are less challenging for dogs with subclinical MMVD and severe LA enlargement or dogs with heart failure. Despite generating 108 data points for each measurement, our study protocol only involved 9 dogs and 2 different operators. More dogs and more operators would have been ideal. Additionally, true LA size could have changed during the study period of 1 week. We contend such a change would be unlikely given the slowly progressive nature of the subclinical stage of this disease in most dogs.^9^, ^10^ Our results are only directly relatable to the echocardiographers (operators) who provided data for this study. Notably, these operators consist of a cardiologist and a resident who works under the supervision of the cardiologist at the same institution. Thus, some degree of conformity bias is likely. To more comprehensively evaluate interoperator variability and avoid conformity bias, more operators from multiple, independent institutions should be involved. Therefore, our results do not necessarily represent all echocardiographers. This design feature is an unavoidable shortcoming of all reproducibility studies.

In conclusion, our study suggests linear measurements of LA size are more reproducible compared to volume estimate measurements. Among the linear measurements, LAD and LAD/AoD exhibited superior reproducibility compared to LA/Ao. Operator was a significant source of variability for LA/Ao, but not for any of the other indices. Although subject to variation depending on operator skill, intraoperator and interoperator 95% RC are available for each of the indices of LA size to help determine if changes in LA size in dogs with MMVD are clinically relevant.

## CONFLICT OF INTEREST DECLARATION

Authors declare no conflict of interest.

## OFF‐LABEL ANTIMICROBIAL DECLARATION

Authors declare no off‐label use of antimicrobials.

## INSTITUTIONAL ANIMAL CARE AND USE COMMITTEE (IACUC) OR OTHER APPROVAL DECLARATION

Approved by the University of California, Davis IACUC, protocol #20438.

## HUMAN ETHICS APPROVAL DECLARATION

Authors declare human ethics approval was not needed for this study.
